# Quantitative Metaproteomic Characterization of Acetic Acid Bacteria Reveals Functional Dynamics During Verdejo Wine Acetification

**DOI:** 10.3390/proteomes14020027

**Published:** 2026-05-20

**Authors:** Cristina Campos-Vázquez, Juan C. García-García, Juan Carbonero-Pacheco, Juan J. Román-Camacho, Roger Consuegra-Rivera, Teresa García-Martínez, Isidoro García-García, Inés M. Santos-Dueñas, Juan Carlos Mauricio

**Affiliations:** 1 Department of Inorganic Chemistry and Chemical Engineering, Chemical Engineering Area, Marie Curie Building (C3), Instituto Químico para la Energía y el Medioambiente (IQUEMA), Agrifood Campus of International Excellence ceiA3, Campus of Rabanales, Universidad de Córdoba, Ctra. N-IV-A, Km 396, 14014 Córdoba, Spain; q42cavac@uco.es (C.C.-V.); isidoro.garcia@uco.es (I.G.-G.); 2 Department of Agricultural Chemistry, Edaphology and Microbiology, Microbiology Area, Severo Ochoa Building (C6), Agrifood Campus of International Excellence ceiA3, Campus of Rabanales, Universidad de Córdoba, Ctra. N-IV-A, Km 396, 14014 Córdoba, Spain; p22gagaj@uco.es (J.C.G.-G.); b12capaj@uco.es (J.C.-P.); mi2gamam@uco.es (T.G.-M.); mi1gamaj@uco.es (J.C.M.); 3 Center for Research and Innovation in Biodiversity and Climate Change (Adaptia), Faculty of Basic and Biomedical Sciences, Universidad Simón Bolívar, Barranquilla 080002, Colombia; z92corir@uco.es

**Keywords:** quantitative metaproteomics, *Komagataeibacter europaeus*, vinegar, verdejo wine

## Abstract

**Background:** Acetification is a complex process driven by acetic acid bacteria (AAB), in which high ethanol and acidity levels require strong microbial metabolic adaptation. Although the microbiota involved in vinegar production has been described, the functional mechanisms that enable these bacteria to maintain metabolic activity remain poorly understood. In this study, the functional dynamics of AAB during Verdejo vinegar acetification were analyzed using a quantitative metaproteomic approach. **Methods:** Acetification was performed in submerged culture under semi-continuous conditions, and samples were collected at four stages of the cycle (S1–S4). **Results:** LC-MS/MS analysis led to the identification of 1626 proteins, of which 1409 were assigned to the Acetobacteraceae family. *Komagataeibacter europaeus* was the dominant species (73.7%). Hierarchical clustering revealed four protein abundance patterns, and differential analysis identified 350 proteins with increased abundance and 169 with decreased abundance, with the greatest changes observed between S1 and S4. Functional annotation and protein–protein interaction analyses indicated that the main metabolic adaptations involve pathways related to energy metabolism, amino acid biosynthesis, membrane-associated functions, cellular homeostasis, and acid stress response. **Conclusions:** Overall, the results show that *K. europaeus* concentrates most of the metabolic activity during acetification and that proteome reorganization reflects key molecular strategies for adaptation and survival under high-acidity conditions.

## 1. Introduction

Vinegar is a traditional fermented condiment widely used in the food industry due to its sensory properties and health benefits [1]. Its production at an industrial level is mainly based on the activity of acetic acid bacteria (AAB), which can partially oxidize ethanol to acetic acid through the combined action of alcohol dehydrogenase (ADH) and aldehyde dehydrogenase (ALDH), with pyrroloquinoline quinone (PQQ) as a coenzyme [2,3]. However, acetic acid may also be toxic to some AAB, which creates a challenge for their survival during the fermentation process. To cope with acetic acid stress, these bacteria have developed several resistance strategies, including membrane remodeling, active efflux systems, intracellular pH homeostasis, molecular chaperones, and oxidative stress response mechanisms [1,4,5,6,7,8,9].

Despite existing knowledge on technical aspects of vinegar-making and basic metabolism of AAB, the specific mechanisms that allow them to survive and maintain their metabolic activity under conditions of high acetic acid and ethanol concentration have not yet been fully elucidated. The lack of a detailed understanding of these processes represents a barrier to optimizing industrial vinegar production and improving the efficiency of the acetification process. In addition, the exact microbial composition of vinegar remains an underexplored area. Although, from a quantitative point of view, AAB have been identified as the main microorganisms involved in acetification, recent studies have shown that the vinegar microbiota is much more complex than previously thought. Typically, genera such as *Acetobacter*, *Gluconobacter*, *Gluconacetobacter*, and *Komagataeibacter* are the AABs found in higher abundance in submerged vinegar culture systems, probably due to their efficient enzymatic ability to transform ethanol into acetic acid [10].

In recent years, omics technologies have enabled deeper insights into the microbial communities involved in vinegar production. Different approaches, including genomics [8,11], transcriptomics [12], proteomics [4,13,14], and metabolomics [15,16,17], have been used to investigate both microbial diversity and the functional processes occurring during acetification. Among these, proteomic and metaproteomic approaches are particularly relevant because proteins directly participate in biological processes and therefore represent a clear indicator of cellular metabolic activity. Consequently, the comprehensive study of the proteome provides an integrated view of the behavior of biological systems and helps to understand how microbial communities respond to environmental changes [18]. In this context, proteomic and metaproteomic strategies have recently been applied to the study of vinegar fermentation, contributing to the elucidation of the adaptive mechanisms that allow acetic acid bacteria to cope with stress conditions such as high ethanol and acetic acid concentrations [5,9,13,19,20,21].

Although previous studies have described the microbial composition of vinegar fermentations, taxonomic profiles alone do not reveal which microorganisms are metabolically active or which pathways are directly involved in adaptation to industrial acetification conditions. In acidic submerged systems, microorganisms are exposed mainly to simultaneous stresses including ethanol, acetic acid accumulation, and oxidative metabolism. Understanding how these factors reshape the active proteome is essential for improving process efficiency, microbiological control, and product quality. In this context, metaproteomics represents a particularly valuable approach because it provides direct information on expressed proteins and ongoing cellular functions within complex microbial communities. This study aims to investigate the functional dynamics of acetic acid bacteria during the acetification of Verdejo wine using a quantitative metaproteomic approach, focusing on protein abundance patterns and metabolic adaptation to the increasing acidity generated during the process. To this end, a quantitative metaproteomic approach using liquid chromatography coupled to tandem mass spectrometry (LC-MS/MS) was employed. The present study follows our previous one, conducted by Campos-Vázquez et al. [10], where the suitability of Verdejo wine as a substrate and the impact of the operating conditions on the microbial communities were evaluated. The properties of Verdejo wine, its availability on the market, and its stability during acetification, together with its popularity as a young white wine in Spain and in other parts of the world, support its potential as a raw material for vinegar production [22,23]. Furthermore, this research also aims in the medium–long term to facilitate the development of innovative strategies for controlling microbiota, allowing the production of vinegars with differentiated characteristics and enhanced added value in the global market. The results highlight the dominant role of *K. europaeus* during the acetification process and reveal key metabolic pathways involved in microbial adaptation to the highly acidic conditions that characterize industrial vinegar production.

## 2. Materials and Methods

### 2.1. Raw Material

The white wine used as a substrate for acetification was made from the Verdejo grape variety and came from the winery “Cooperativa La Unión” (Montilla, Córdoba, Spain). Initially, it presented an ethanol concentration of 101.0 ± 0.8 g/L, and was diluted with distilled water until the optimum working conditions were reached, obtaining a final ethanol content of 78.9 ± 0.8 g/L. As for its chemical composition, the medium contained a total acidity of 5.3 ± 0.1 g/L, a volatile acidity of 0.2 ± 0.1 g/L, and a concentration of reducing sugars of 1.0 ± 0.1 g/L.

### 2.2. Microorganisms

For the preparation of the working inoculum for acetification, 500 mL of an industrial fully active fermentation medium collected from a working tank of the company UniCo Vinagres y Salsas, S.L.L. (Doña Mencía, Córdoba, Spain), was used. This inoculum consisted of the resident mixed microbial consortium naturally present in the industrial acetification system. In addition, 1 L of fine wine vinegar and a modified synthetic alcoholic medium were added to the inoculum [21].

### 2.3. Fermentation Conditions

The acetification process was carried out in a fully automated 8 L Frings bioreactor (Heinrich Frings GmbH & Co., KG, Bonn, Germany), operating in a semi-continuous mode. The operating conditions were a constant temperature of 31 °C, a continuous air supply of 7.5 L/(h L of medium), and a feed rate of 1.3 ± 0.1 L/h. A total of 35 adaptation and calibration cycles were performed prior to 25 stable cycles used for sampling, with an average duration of approximately 22 h per cycle.

### 2.4. Sampling

To monitor the evolution of the microbiota, samples were collected at four specific time points during the cycle: at the end (S1) of the loading stage, corresponding to the highest ethanol concentration (33.1 g/L), and subsequently at three key moments during its depletion in the medium: 23.7 g/L (S2), 15.8 g/L (S3), and 7.9 g/L (S4), the latter just before unloading. Four biological replicates were obtained for each sampling point (*n* = 4), yielding 16 samples in total. Additionally, one inoculum sample and one raw material sample were included, giving a total of 18 samples.

### 2.5. Analytical Methods

System variables were continuously monitored throughout the process. Reactor volume was measured using an EJA 110 differential pressure probe (Yokogawa Electric Corporation, Tokyo, Japan), ethanol concentration was determined with an Alkosens R probe (Heinrich Frings GmbH & Co., KG, Bonn, Germany), and temperature was recorded using a temperature probe. The automated control system allowed continuous data acquisition, ensuring stable monitoring conditions and confirming the high reproducibility of the experimental method. Total acidity (g/L) was determined by acid-base titration using 0.5 N NaOH. In addition, total cell concentration was quantified at each sampling point by optical microscopy using a Neubauer counting chamber, following the procedure described by Baena-Rueano et al. [24]. Process efficiency was evaluated via the average acetification rate (*r_A_*) and total acetic acid production (*p_A_*), as described by García-García et al. [25].

### 2.6. Metaproteomics

#### 2.6.1. Cell Harvesting and Protein Extraction

Each sample was taken directly from the reactor and divided into four 50 mL Falcon™ tubes (Eppendorf AG, Hamburg, Germany), kept in an ice bath. The cells were recovered by a first centrifugation at 4600× *g* for 10 min at 4 °C (Hettich, Rotina 38R; Westphalia, Germany). The pellets obtained were washed twice with cold, sterile distilled water and, after homogenization, the cells were subjected to two additional centrifugations at 16,900× *g* for 1 min at 4 °C (Eppendorf 5418 R; Hamburg, Germany). The resulting cell sediments were resuspended in 600 μL of extraction buffer, composed of 100 mM Tris-HCl (pH 8.0), 2 mM DTT, and 1 mM EDTA, supplemented with a protease inhibitor cocktail (Roche, Basel, Switzerland). For cell disruption, an equivalent volume of glass beads (Sigma-Aldrich, St. Louis, MO, USA) was added and alternating vortex cycles (ten cycles of 1 min of shaking and 1 min on ice) were applied, followed by sonication in an ultrasonic bath (J.P. SELECTA^®^, Ultrasons, Barcelona, Spain). After lysis, the beads and cell debris were removed by two successive centrifugations: first at 7000× *g* for 10 min at 4 °C, and then at 16,000× *g* for 20 min at 4 °C. In this protocol, protein precipitation using TCA-acetone-DTT was not performed, as this is a highly aggressive method that can compromise the stability and recovery of certain proteins. Instead, the clean supernatants were directly subjected to a solubilization step, resuspending the proteins in 600 μL of solubilization buffer (8 M urea, 2% CHAPS, and 20 mM DTT). This mixture was shaken in four cycles of 30–45 min at 1200 rpm and 4 °C (Heidolph™, Vibramax 100; Schwabach, Germany). Finally, the samples were centrifuged at 16,000× *g* for 15 min at 4 °C, and the protein concentration of the supernatant was determined using the Bradford [26] method [10,19,20,21,27].

#### 2.6.2. LC-MS/MS Analysis

For proteomic analysis, 50 μg of protein per sample was injected into the LC-MS/MS equipment of the Central Research Support Service (SCAI) at the University of Córdoba. Prior to analysis, the samples were cleaned using one-dimensional SDS-PAGE employing a 10% polyacrylamide gel, applied at 100 V to allow the proteins to enter the gel. To initiate digestion, the protein bands were denatured using 200 mM ammonium bicarbonate (AB) in the presence of 50% acetonitrile for 15 min, followed by a brief treatment with 100% acetonitrile for 5 min. The proteins were then reduced with 20 mM dithiothreitol in 25 mM AB for 20 min at 55 °C and alkylated with 40 mM iodoacetamide in 25 mM AB for 20 min in the dark. After this step, the gels were washed twice with 25 mM AB. Enzymatic digestion was performed by adding trypsin (Promega, Madison, WI, USA) at 12.5 ng/μL in 25 mM AB, incubating the samples overnight at 37 °C. The process was stopped by adding 1% TFA, and the peptide digests were concentrated in a SpeedVac™. Chromatographic analysis was performed on a Dionex Ultimate 3000 nano-UHPLC system (Thermo Fisher Scientific, Waltham, MA, USA). The peptides were initially concentrated on an Acclaim PepMap C18 precolumn (5 mm × 0.3 mm) with a flow rate of 5 μL/min for 5 min in a mixture of 2% acetonitrile/0.05% TFA. Separation was then performed on an Acclaim PepMap C18 analytical column (500 mm × 75 μm) at 40 °C, applying a gradient of 4–90% acetonitrile/0.1% formic acid for 150 min, with a flow rate of 300 nL/min. Detection was performed using an Orbitrap Fusion (Thermo Fisher Scientific, Waltham, MA, USA) equipped with nano-electrospray. MS spectra were acquired between 400–1500 *m*/*z*, with a resolution of 120,000 (at 200 *m*/*z*) and a target of 4 × 10^5^ ions. For MS/MS, precursors selected by the quadrupole (1.2 Th isolation window) were fragmented using CID with a normalized collision energy of 35%, and the fragments were analyzed in the ion trap in fast scan mode. The AGC target for MS/MS was 2 × 10^3^ with a maximum injection time of 75 ms. Only precursors with charge states +2 to +5 were selected for fragmentation, applying a 15 s dynamic exclusion with a tolerance of 10 ppm. Acquisition was set to “top speed” mode, operating in 3 s cycles, during which the equipment executed as many MS/MS events as possible until the list of available precursors was exhausted [10,19,20,21,27].

#### 2.6.3. Data Analysis

Protein raw data were filtered according to the criteria previously established in studies from our research group and following the approach described by Campos-Vázquez et al. [10], including protein frequency thresholds. These filtering steps were applied to remove low-confidence identifications and improve the robustness of the dataset [19,20,21]. Specifically, proteins with a score ≤ 2 or supported by fewer than two peptides were discarded, and only those classified with “High” FDR confidence were retained for further analysis. Taxonomic classification was based on the UniProt database (http://www.uniprot.org, accessed on 2 March 2026). Enrichment analysis was carried out using the Gene Ontology (GO) annotation tool (http://geneontology.org, accessed on 2 March 2026). Functional visualization was performed with heatmaps using R (v4.4.1). Protein values were normalized by dividing each one by the sample global intensity from all samples and then centered by z-score transformation. Intersection analysis was conducted with the “Upset” R library, using proteins present in ≥50% out of samples. For the hierarchical clustering and heatmap analysis, proteins identified in at least 50% of samples in each sampling time were used. One-way ANOVA followed by HSD Tukey’s test was calculated by R packages (v4.4.1). Network construction and visualization were performed using Cytoscape software (v3.10.2), where proteins were represented as nodes connected to their corresponding GO terms, allowing the identification of functional patterns associated with differentially abundance proteins. For the analysis of the significant proteins (*p*-value < 0.05), protein–protein interaction analysis was performed using the STRING v12.0 database (https://string-db.org, accessed on 2 March 2026), with *Komagataeibacter europaeus* as a search model. The interaction of maximum confidence (score *>* 0.90) was applied. Nodes were grouped in colors based on protein annotations according to the databases UniProt and KEGG, both available in STRING. Protein identification was based on high-resolution LC-MS/MS data and matched against the UniProt database. Given that the main microorganisms involved in vinegar acetification are well represented in curated databases, protein identification relied on homology-based annotation rather than de novo sequencing or isolation. Taxonomic assignment was interpreted considering potential ambiguity from conserved proteins, prioritizing high-confidence identifications supported by multiple peptides.

## 3. Results and Discussion

### 3.1. Description of System Variables

**Figure 1:**
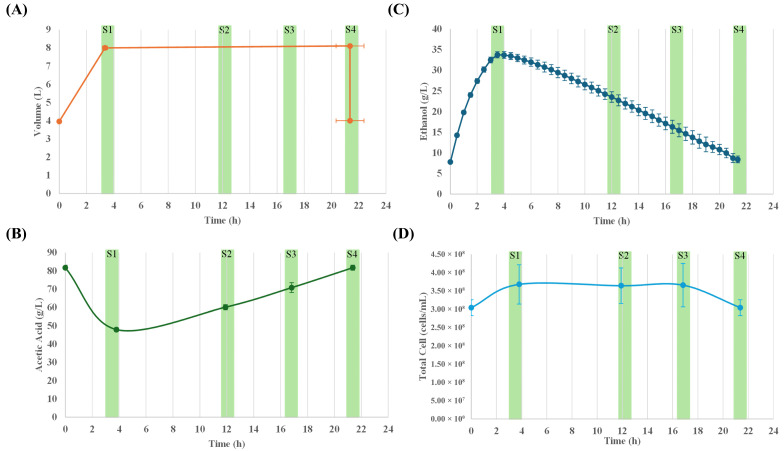
Main system variables throughout the acetification profile of Verdejo wine, including both the variables continuously monitored and those measured exclusively at sampling times: (**A**) working volume, (**B**) acetic acid concentration, (**C**) ethanol concentration, and (**D**) total cell count. Green shaded areas indicate the sampling stages (S1–S4). The average values of these variables in each sample are presented together with their corresponding standard deviations (SD).

Following the methodology indicated in the Materials and Methods section, 35 adaptation and calibration cycles were carried out before starting the 25 stable cycles for sampling. The average time of each cycle was approximately 22 h. Figure 1 shows the profile of some variables during acetification. Through the loading phase, the reactor volume and ethanol concentration increased rapidly, reaching a maximum volume of 8.0 ± 0.1 L and an ethanol concentration of 33.1 ± 0.8 g/L (S1) within 3 h. Subsequently, maintaining the volume of medium in the reactor constant, the depletion phase begins, and when an ethanol concentration of 8.7 ± 0.1 g/L (S4) is reached, 50% of the fermenter’s contents (4 L) is unloaded so that a new cycle can begin immediately. During the depletion phase, acidity experienced a significant increase from 48.0 ± 1.0 to 82.0 ± 1.0 g/L, while total cell concentration peaked at 3.7 ± 0.5 × 10^8^ cells/mL, remaining stable until approximately 16.5 h, then decreasing slightly to 3.0 ± 0.2 × 10^8^ cells/mL. Ethanol concentration at unloading time, fraction of medium removed, and loading rate are operational variables that influenced the acetification profile.

### 3.2. Qualitative Analysis: Microbial Composition

**Figure 2:**
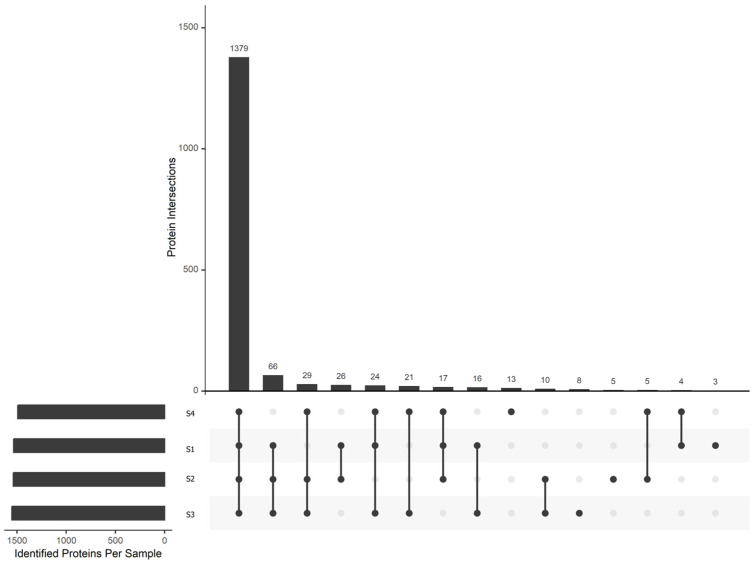
Intersection diagram displaying the proteins identified in at least 50% of the total samples in at least one sampling time. The bars represent the number of proteins corresponding to each intersection group.

**Figure 3:**
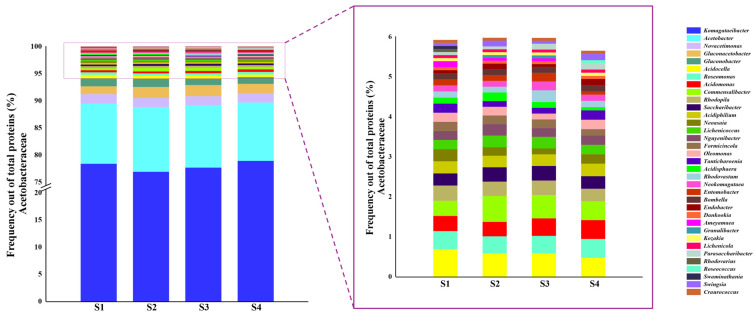
Bar plot represents the protein frequency (%) of each identified genus within the family Acetobacteraceae, calculated using only the proteins assigned to this family (*n* = 1409) for each sampling point (S1, S2, S3, and S4). The inset panel shows a magnified view of the low-abundance genera for clarity.

The metaproteomic analysis (LC-MS/MS) identified 1626 valid proteins across the acetification profile, originating from 149 species that comprise the metaproteome from the microbiota of Verdejo wine vinegar. The proteins identified in at least ≥50% of the replicates and at least one sampling point, have been plotted in an intersection graph (Figure 2). Of these, according to the selection criteria of common proteins, those present in ≥50% of the biological replicates at each sampling time, 1489 proteins were identified out of those of the acetification profile. Among these common proteins, 1409 (94.5% of total common proteins) were assigned to the family Acetobacteraceae, confirming the predominance of acetic acid bacteria during the acetification process. In addition, Figure 3 shows the taxonomic distribution of genera considering only the proteins assigned to Acetobacteraceae (*n* = 1409). Within this family-level subset, *Komagataeibacter* was the predominant genus, followed by *Acetobacter*, whereas other minor genera such as *Gluconacetobacter* (1.7%), *Novacetimonas* (1.6%), and *Gluconobacter* (1.3%) were also detected among those with frequencies ≥1%. Based on the total set of common proteins (*n* = 1489), *Komagataeibacter europaeus* was the most represented species, accounting for 73.7%, followed by *Acetobacter aceti* (10.7%). The complete list of valid proteins identified can be seen in Table S1 of the Supplementary Materials.

The dominance of *K. europaeus* could be attributed to its higher ability to tolerate stress conditions imposed by the process, including high concentrations of acetic acid and ethanol. This species is known for its efficient metabolic capacity, based on pathways such as ethanol oxidation by specific and stable dehydrogenases complexes, as well as its ability to maintain high energy stability through proton gradient formation and ATP generation [3]. These results agree with previous studies on the microbiological characterization of Verdejo wine vinegar [10], which highlighted the importance of AAB as the central functional group in acetification.

### 3.3. Hierarchical Clustering: Grouping of Proteins by Quantification Patterns

**Figure 4:**
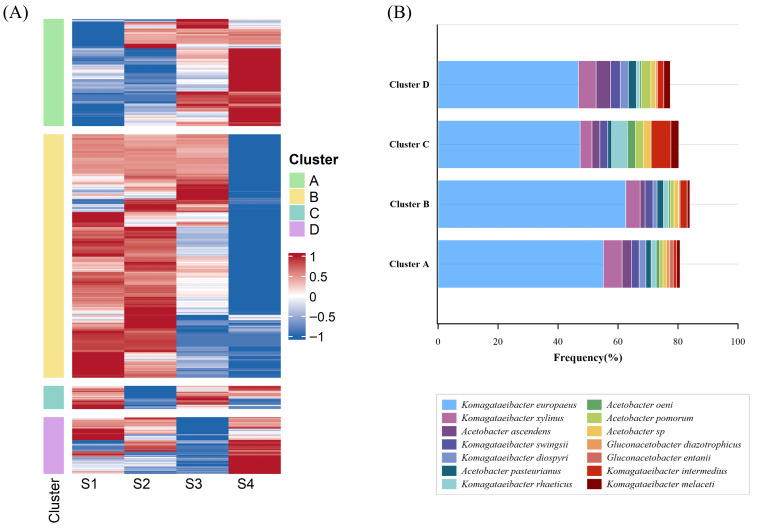
(**A**) Heat map showing the analysis of hierarchical clustering of the Acetobacteraceae proteins identified in at least 50% of total samples in each sampling time (S1, S2, S3, and S4). The letter on each color represents each of the four built clusters. (**B**) Relative abundance of the Acetobacteraceae species mostly detected in each cluster during the acetification process (>1%).

Hierarchical clustering revealed specific protein quantification patterns across sampling points (S1–S4). Common proteins of Acetobacteraceae were grouped based on their quantification profile. For this purpose, the quantification value of each protein was normalized by z-score transformation and then grouped according to its pattern. Figure 4A presents a heatmap of the hierarchical clustering of 1409 proteins into four clusters. Full protein lists and annotations are provided in Table S2, in Supplementary Materials.

Four distinct protein clusters (A–D) were identified, each displaying a characteristic abundance pattern across the acetification stages.

- **Cluster A (*n* = 351):** Contains more proteins abundant at the final sampling time (S4), associated with a low ethanol concentration and high acidity values. Here, 73.50% of proteins belonged to *Komagataeibacter* genus, and the main species were *K. europaeus* (55.11%), *Komagataeibacter xylinus* (6.26%), and *Acetobacter ascendens* (3.12%).
- **Cluster B (*n* = 797):** Consists mainly of proteins present in the early moments of the cycle, when there is a high concentration of ethanol, S1 and S2, just after the loading stage is complete. *Komagataeibacter* was the most abundant genus (76.63%), and the species *K. europaeus* (62.61%), *K. xylinus* (4.77%), and *Komagataeibacter swingsii* (2.63%) dominated this group.
- **Cluster C (*n* = 76):** This cluster presented mixed protein profiles (S1-S3-S4). Most proteins belonged to *Komagataeibacter* genus (77.63%), and for the species the most abundant were *K. europaeus* (47.77%), *Komagataeibacter intermedius* (6.58%), *Komagataeibacter rhaeticus* (5.26%), and *K. xylinus* (3.95%).
- **Cluster D (*n* = 186**): Like Cluster C, this cluster presented mixed protein profiles, with a stronger association with S4. Following the trend of the other clusters, *Komagataeibacter* was the predominant genus (77.63%). *K. europaeus* (46.77%), *K. xylinus* (5.91%), *A. ascendens* (4.84%), *Acetobacter pomorum* (3.22%), and *K. swingsii* (3.22%) were the dominant species.

As shown in Figure 4B, the species within each cluster that accounted for more than 1% of the total protein content within their respective cluster were plotted. Collectively, these species account for approximately 80% of the protein profile of each cluster. In all four cases, the dominant species belonged mainly to the genera *Komagataeibacter* and *Acetobacter*, which is consistent with various studies indicating that these genera are primarily responsible for the acetification process in both industrial systems and traditional processes, as well as in the early stages of submerged culture acetification [21,28,29,30].

Regarding the predominant species, it was observed that *K. europaeus* increased in abundance during the initial and intermediate stages of the acetification process, subsequently decreasing slightly as the cycle progressed, reaching its lowest value in S4. This species has been described by numerous authors as one of the most abundant in industrial fermentation processes [21,31,32]. This pattern reinforces the central role of *K. europaeus* in acetification, due to its high tolerance to ethanol and acid stress, as well as its high capacity for acetic acid production [10,21,33].

### 3.4. Statistical Analysis and Differential Abundance: Significant Proteins

Proteins with differential abundance across sampling points were identified by one-way ANOVA, followed by Tukey’s post hoc test. Proteins showing significant variation (*p* < 0.05) were selected for further analysis.

**Figure 5:**
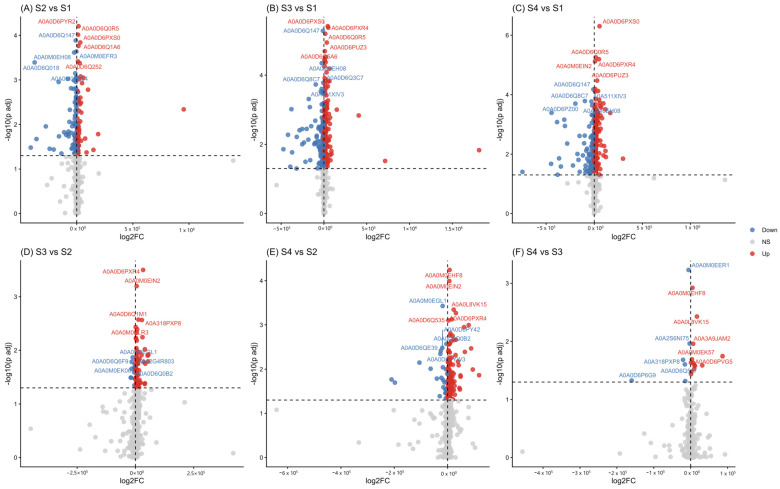
Volcano plots showing the differential protein abundance between sampling points during the acetification cycle. Comparisons correspond to (**A**) S2 vs. S1, (**B**) S3 vs. S1, (**C**) S4 vs. S1, (**D**) S3 vs. S2, (**E**) S4 vs. S2, (**F**) S4 vs. S3. The x-axis represents the log_2_ fold change (log2FC), while the y-axis indicates the statistical significance (−log_10_ *p*-value). Proteins with significant differences (*p* < 0.05) are highlighted, proteins with increased abundance were shown in red and proteins with decreased abundance in blue, whereas non-significant proteins are represented in gray.

A total of 350 proteins with increased abundance and 169 proteins with decreased abundance were identified. Post hoc tests were performed using Tukey’s test corrected by multiple testing (*q* < 0.05); marked changes were highlighted between sampling points S1–S4 and S2–S4 (see Table S3 in Supplementary Materials). These differences reflect the activation and repression of key metabolic pathways throughout the process (Table S4 Supplementary Materials). The highest number of proteins with increased abundance was recorded in the final sampling times (S3 and S4). In the volcano plot (Figure 5), the proteins with the most significant changes (*log2FC* > 1 or < −1) are highlighted, allowing the identification of those that could play key roles in the metabolic adaptation of the system. Taken together, these results indicate that the largest number of significant proteins and the highest fold changes were observed in comparisons involving S4, indicating a stronger metabolic shift in the late stages of the acetification process. In contrast, comparisons between earlier stages (S2–S1 and S3–S2) showed fewer significant proteins, suggesting a more stable proteomic profile during the initial phases.

**Figure 6:**
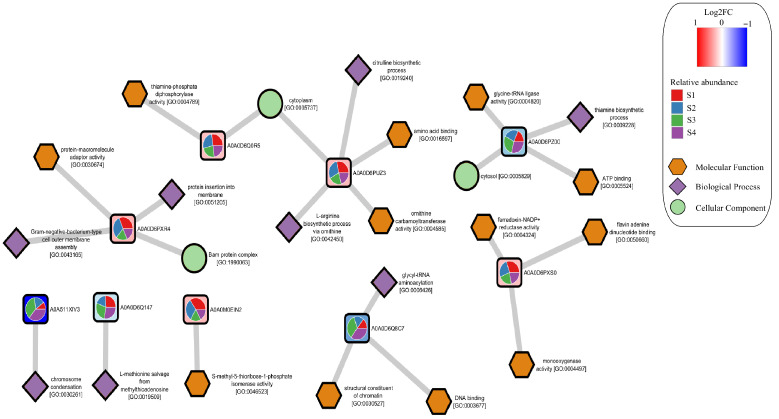
Network representation of the association between significant proteins and their corresponding Gene Ontology (GO) terms during the acetification process. Proteins showing significant differences (*p* < 0.05) between S1 and S4 were included in the analysis. Nodes represent proteins and GO terms, while edges indicate functional associations based on UniProt annotations. Protein nodes are colored according to their log_2_ fold change (log2FC, calculated as S4 vs. S1), indicating proteins with increased abundance in S4 relative to S1 (red) or decreased abundance in S4 relative to S1 (blue). Pie charts within protein nodes represent the relative abundance across sampling points (S1–S4). GO terms are grouped according to their functional category: Molecular Function (orange hexagons), Biological Process (green circles), and Cellular Component (purple diamonds).

To evaluate the functional changes associated with the progression of acetification, the set of the 10 proteins showing the most significant changes in the comparison between S1 and S4 was selected, as these sampling points represent the beginning and the end of the acetification process, respectively. Using these proteins, a protein-GO term association network was constructed (Figure 6). All the proteins with increased abundance between S1 and S4 belonged to *K. europaeus* (highlighted in red in Figure 6), highlighting the predominant role of this species during the acetification process [10,21]. These proteins were mainly associated with processes related to amino acid metabolism, membrane assembly, and redox reactions, including enzymes involved in arginine biosynthesis and membrane protein insertion. These functions are consistent with the physiological adaptations required during acetification, where the cell membrane plays a fundamental AAB, functioning as one of the key mechanisms for stress regulation [34]. Moreover, the membrane also plays a crucial role in acetic acid production in AAB, since this process occurs in the periplasmic space [19,35]. Altogether, these results suggest that *K. europaeus* concentrates most of the metabolic activity required to sustain both cellular growth and the conversion of ethanol into acetic acid under stress conditions.

The functional nature of the proteins increased abundance in this study, mainly associated with amino acid metabolism, membrane assembly/structure, and redox reactions, including enzymes involved in arginine biosynthesis and membrane protein insertion, is consistent with quantitative studies specifically focused on *K. europaeus*. In these studies, it has been reported that at the end of the loading phase, the predominant functions in this species include amino acid metabolism, energy pathways, redox activity, and the presence of multiple outer membrane proteins, such as ABC transporters and porins, unequivocally identified in its proteome. The increased abundance of enzymes related to glutathione reductase, N-succinyl-transferases, and aminoacyl-tRNA ligases, together with proteins involved in redox balance maintenance (thioredoxins, PQQ-dependent dehydrogenases, and oxidative stress response proteins), suggests a coordinated metabolic reprogramming of *K. europaeus* aimed at maximizing energy efficiency and protection against reactive oxygen species generated during acetification [19,20]. The enrichment of proteins related to arginine biosynthesis may reflect an increased demand for nitrogen-containing metabolites during the late stages of acetification. Amino acid availability has been shown to influence metabolism and acid stress resistance in acetic acid bacteria, supporting the relevance of amino acid-related pathways under these conditions [9,35].

In contrast, the analysis of proteins with decreased abundance (highlighted in blue in Figure 6) between S1 and S4 also showed a predominance of *K. europaeus*, although two proteins belonging to *Acetobacter oeni* and *K. xylinus* were detected. These proteins were associated with functions related to vitamin biosynthesis, amino acid recycling, protein synthesis, and chromosome organization, including processes such as thiamine biosynthesis and tRNA aminoacylation. In transcriptomic studies of acetic acid bacteria during high-acidity submerged fermentation, down-regulated genes are consistently enriched in ribosomal proteins and translation machinery (including aminoacyl-tRNA ligases), reflecting an inhibition of protein synthesis as an early response to acid stress [4,13,36].

**Figure 7:**
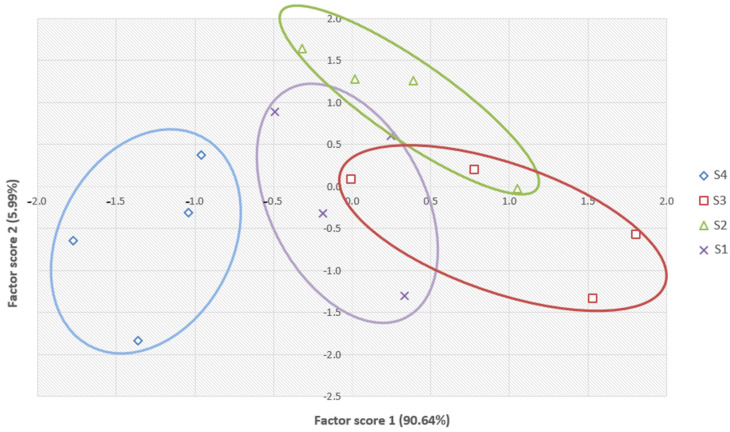
Principal Component Analysis (PCA) of differentially significant proteins. The figures and colors indicate the sampling points represented (S1, S2, S3, and S4). The ellipses represent the variability within each group.

Finally, Principal Component Analysis (PCA) (Figure 7) revealed a clear separation among S1–S4 times, indicating biological differences in protein quantification dynamics throughout the vinegar-making process.

### 3.5. Associated Functions of Significant Proteins

#### 3.5.1. GO Term Enrichment Analysis

A GO Term enrichment analysis was carried out on the proteins that showed significant differences during the acetification process, for the Biological Processes, Cellular Component, and Molecular Function categories at four sampling points (S1 to S4). Heatmaps were used with the aim of understanding the functions involved in acetification. These plots are based on the relationship found between the Acetobacteraceae microorganisms detected and the GO Terms of each category according to the quantification of the protein, using the UniProt database.

##### Biological Process

**Figure 8:**
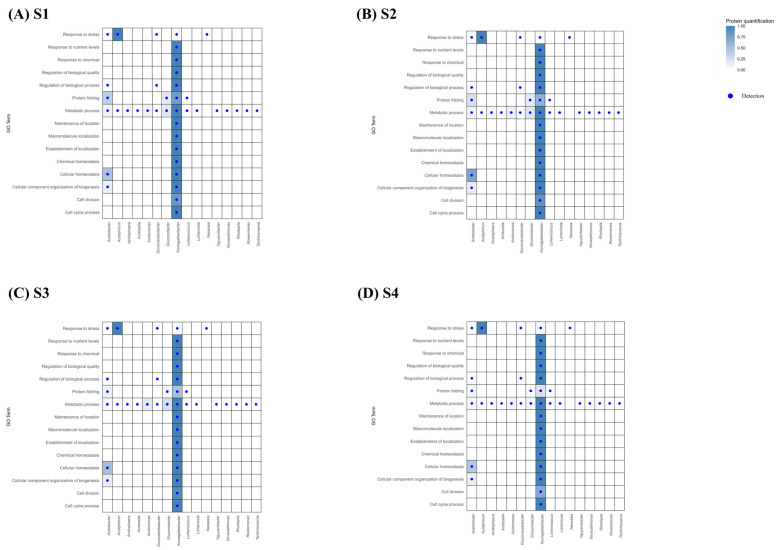
Heatmap showing the relationship among the microorganisms of Acetobacteraceae family detected in the acetification process ((**A**) S1, (**B**) S2, (**C**) S3, (**D**) S4) and the GO Terms in the Biological Processes category with which these microorganisms are related in terms of protein quantification.

For the Biological Processes category (Figure 8), proteins were mainly related to the functions “Metabolic processes” (S1: 7.88%, S2: 7.98%, S3: 8.61%, S4: 7.37%), “Cellular homeostasis” (S1: 7.88%, S2: 9.30%, S3: 8.38%, S4: 8.43%), “Cell division” (S1: 9.78%, S2: 10.04%, S3: 10.78%, S4: 9.84%), and “Protein folding” (S1: 14.2%, S2: 12.11%, S3: 9.77%, S4: 8.06%), these being the major processes at all sampling points, indicating a highly stable functional dynamic. However, within these functional terms, different patterns were identified throughout the acetification cycle, with “Protein folding” predominating in S1 (14.22%) and S2 (12.11%), while “Cell division” was more relevant in S3 (10.78%) and S4 (9.84%). These changes are mainly influenced by environmental conditions, such as ethanol concentration, pH, and substrate availability, which modulate protein abundance of the microorganisms present throughout acetification [37].

*Komagataeibacter*, which dominated most GO Terms (see Figure 8), showed consistently low levels of “Stress response” (<0.25), suggesting an intrinsic tolerance to ethanol and acidic conditions without the need for strong activation of defense mechanisms [5,35]. In contrast, proteins related to “Protein folding” were more abundant in S1 (0.77) and S2 (0.61) and decreased progressively towards S3 (0.49) and S4 (0.22) (Figure 8), suggesting that in these initial moments there is a high activity of protein synthesis and need for structural stabilization. A similar trend to the decrease in “Protein folding” was observed in *Acetobacter*, although with lower overall values (S1: 0.36, S2: 0.23, S3: 0.10, S4: 0.06), see Figure 8. In contrast, the “Response to stress” category in the *Acidiphilium* genus remains high and constant throughout the process (S1–S4: 1), unlike aforementioned genera, suggesting that *Acidiphilium* may experiences sustained acid stress and therefore continuously activates stress-response mechanisms during acetification [38].

Proteins associated with the “Metabolic process” were detected in a wide range of microorganisms; however, *Komagataeibacter* was the only genus that showed the highest intensity for this GO Term, consistent with its role as the main metabolic driver of ethanol oxidation during acetification. Furthermore, “Cellular homeostasis” and the “Cellular components organization of biogenesis” were more represented in *Komagataeibacter* than in *Acetobacter*, suggesting more robust internal regulation and sustained cellular maintenance under process conditions [19,39].

##### Cellular Component

**Figure 9:**
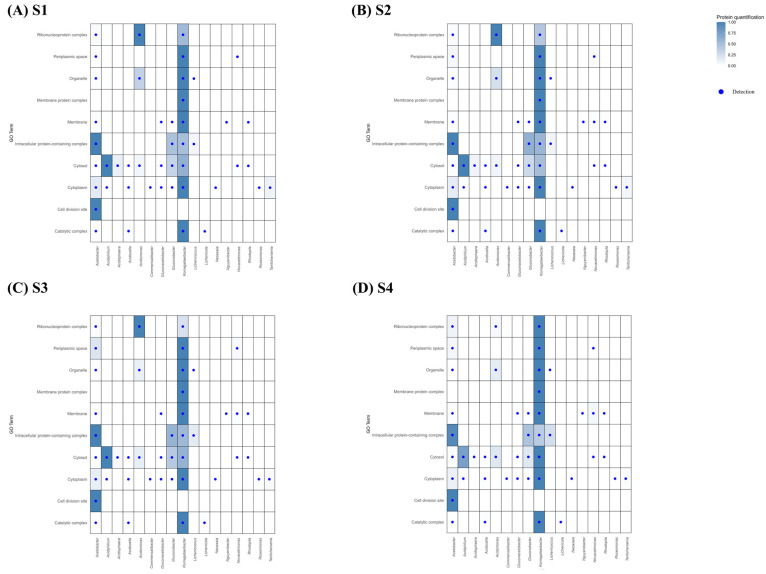
Heatmap showing the relationship among the microorganisms of Acetobacteraceae family detected in the acetification process ((**A**) S1, (**B**) S2, (**C**) S3, (**D**) S4) and the GO Terms in the Cellular Component category with which these microorganisms are related in terms of protein quantification.

In Cellular Component category (Figure 9), there is a clear prevalence of proteins related to “Cytoplasm” (S1:9.91%, S2: 9.04%, S3: 9.01%, S4: 8.91%), “Cytosol” (S1: 14.81%, S2: 13.76%, S3: 15.58%, S4: 15.61%), “Intracellular protein-containing complex” (S1:19.58%, S2: 21.61%, S3: 21.13%, S4: 21.72%), “Ribonucleoprotein complex” (S1:10.81%, S2: 10.16%, S3: 8.78%, S4: 8.16%) and “Organelle” (S1: 9.45%,S2: 8.81%, S3:7.93%, S4: 8.27%), with “Intracellular protein-containing complex” being the most represented throughout the whole process. The function of the “Periplasmic space” presented a higher quantification in *Komagataeibacter*, reaching a value of 1 constantly (S1–S4, see Figure 9). It has been demonstrated that *K. europaeus* possesses specialized periplasmic transport mechanisms that allow it to regulate the metabolites flow in response to the concentration of substrate (ethanol) and product (acetic acid), ensuring greater cellular stability during fermentation [37,40]. The fact that *Komagataeibacter* maintains this GO Term at the maximum level throughout the entire process indicates sustained periplasmic involvement, consistent with stable functional activity despite the changes and variations that occur given under the established acetification working conditions. In contrast, other AABs such as *Acetobacter* and *Novacetimonas* maintained the “Periplasmic space” GO Term at much lower levels than *Komagataeibacter*, which could be related to the dominance of the latter in submerged culture vinegar-making. Moreover, it should be noted that acetic acid synthesis in AAB mainly takes place in the periplasmic space, where key membrane-bound enzymes involved in ethanol oxidation are located. In this context, the GO term analysis indicates that the major contribution to acetic acid production is associated with *Komagataeibacter*, not only due to its predominance in terms of protein quantification, but also because of its strong functional association with the “Periplasmic space” GO term.

On the other hand, *Komagataeibacter* proteins associated with “Cytosol” GO Term exhibited a gradual increase (S1: 0.61–S4: 1) throughout the cycle. During the intermediate stages, periods in those samples S2 and S3 were taken and, especially in the final stage, the moment in which sample S4 was taken, in this genus, a light increase in proteins in “Cytosol” is observed (0.50, 0.60, and 1 respectively), which could be related to a greater activity in the cell inner, possibly, due to the need to process metabolites generated when external conditions do not encourage such a competitive attitude [19,20,21,41,42]. Moreover, “Ribonucleoprotein complex” showed a significantly higher quantification in S4 (0.53), which might reflect the sustained activity of the translational machinery under our working conditions, ensuring protein synthesis and repair at the final stage. This is consistent with the physiology of *K. europaeus*, which dominates the process and is known to maintain intracellular homeostasis, including a slightly acidic cytoplasmic pH, even in the presence of high extracellular acetic acid levels [43]. There is a clear functional divergence between *Komagataeibacter*, which increases the quantification of these complexes in the final phase, and *Acidomonas*, which reduces them, along with the limited involvement of *Acetobacter* in this type of process throughout the acetification cycle. Because the most abundant and prevalent (S1–S4) GO Terms in *Acetobacter* have been “Intracellular protein-containing complex”, “Cytoplasm”, and “Cell division site” see Figure 9, its role could be related with the metabolite exchange with the external environment, possibly because as the cycle progresses, the competitiveness among microorganisms gradually diminishes. For the term “Cell division site,” the absence of *Komagataeibacter* (see Figure 9) again indicates that its role in the process is not associated with high proliferative activity. The high level in *Acetobacter* suggests greater cell division activity, independent of the main function of ethanol oxidation. This reinforces the idea of the dominance of *Komagataeibacter* in acetic acid production by submerged culture compared to *Acetobacter* [19,42].

On the other hand, the decrease observed for “Organelle” in *Acidomonas* suggests a gradual loss of cellular activity as acetification progresses. This finding is consistent with the decline in other indicators of lower functional activity in *Acidomonas* in the final stages. Regarding *Acidiphilium*, the high quantification level from the start indicates sustained metabolic activity, although with some reduction at the end of the process.

##### Molecular Function

**Figure 10:**
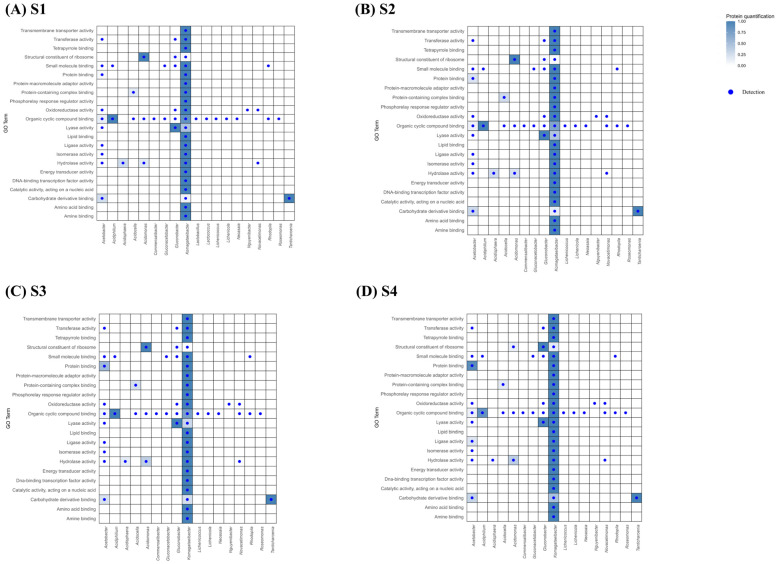
Heatmap showing the relationship among the microorganisms of Acetobacteraceae family detected in the acetification process ((**A**) S1, (**B**) S2, (**C**) S3, (**D**) S4) and the GO Terms in the Molecular Function category with which these microorganisms are related in terms of protein quantification.

In the Molecular Function category (Figure 10), GO Terms associated with “Carbohydrate derivative binding” (S1: 9.03%, S2: 9.26%, S3: 9.32%, S4: 9.91%), “Hydrolase activity” (S1: 5.66%, S2: 5.81%, S3: 5.78%, S4: 5.13%), “Lyase activity” (S1: 5.66%, S2: 5.55%, S3: 4.95%, S4: 7.04%) and “Organic cyclic compound binding” (S1: 8.64%, S2: 9.26%, S3: 7.86%, S4: 8.39%) stand out, with all of them present at the total acetification sampling times. Of these, “Carbohydrate derivative binding” was the most abundant, reflecting the importance of interactions with carbohydrate derivatives in the functionality of the microbiota. However, in S3 and S4, there was a greater representation of “Protein binding” (5.80% and 6.73%, respectively), which indicates a change in functional activity as fermentation progresses.

Throughout the cycle, dynamic quantification changes were detected of these molecular functions for the different microorganisms. A constant significant increase in “Protein binding” was observed in *Acetobacter* (S1: 0.05, S2:0.21, S3:0.54, S4: 0.91), increasing to values close to 1, accompanied by an increase in “Ligase activity” (S1: 0.01, S2: 0.01, S3: 0.03, S4: 0.20). In other studies, it was seen that protein synthesis would tend to increase under high acid stress in *A. pasteurianus* [13]. This could occur in our study, where the highest values of these functions were detected at the final moment (S4). The increase in *Acetobacter* suggests greater progressive functional complexity, possibly associated with cellular adjustments throughout the process as acidity level increases in the medium. In contrast, *Komagataeibacter* maintains a high and constant level of “Ligase activity”, which could indicate stable functional architecture, with protein interactions maintained regardless of environmental conditions. This would reinforce the idea of *Komagataeibacter’s* robustness in the face of high acidity conditions and its dominance of the acetification process by these media over *Acetobacter*. Regarding *Acidomonas*, a considerable proportion of proteins related to “Structural constituent of ribosome” was maintained throughout the process, until a considerable decrease was experienced (S1–S3: 1, S4: 0.12), indicating a lower ribosomal activity in this final time, possibly influenced by the stress characteristics of this period. In contrast, in the case of AABs such as *Gluconobacter* and *Komagataeibacter*, the opposite occurred, and levels increase throughout the process for this Go Term [42].

The sustained high levels of “Organic cyclic compound binding” in *Acidiphilium* (S1–S3: 1, S4: 0.86) suggest greater involvement in interactions with this type of compound throughout the process. Known for metabolizing aromatic compounds under acidic conditions [38], *Acidiphilium* gains an adaptive advantage by binding and processing these compounds, ensuring its stability and functionality throughout acetic acid fermentation. In *Komagataeibacter*, “Organic cyclic compound binding” increased in the final time (S1: 0.86, S2: 0.71, S3: 0.75, S4: 1), along with “Lyase activity” (S1: 0.47, S2: 0.45, S3: 0.30, S4:1), both reaching values of 1. This suggests that *Komagataeibacter* might use these strategies to modify or degrade substrates at the final moments of the process. It may shift its metabolism to assimilate alternative carbon sources when ethanol becomes scarce [37,44].

Meanwhile, *Tanticharoenia* consistently showed high levels of proteins related to “Carbohydrate derivative binding” (S1–S4: 1, see Figure 10). In the final stages, as acetic acid levels rise and pH decreases, its interaction with carbohydrate derivatives may be linked to exopolysaccharides (EPS) synthesis or modification for acid stress resistance; many AABs produce EPS as a protective response to hard conditions [45]. *Tanticharoenia* showed consistently high levels of proteins related to this function throughout acetification. This behavior might indicate greater sensitivity to environmental stress, leading to the maintenance of EPS-related functions from the initial stages to ensure cell protection in increasingly adverse conditions. Unlike *Tanticharoenia*, *Komagataeibacter* showed low levels of this function during the initial times, followed by a slight increase at the end. This behavior could support the idea that *Tanticharoenia* is more sensitive to environmental stress than other species of AABs.

### 3.6. Analysis of Protein–Protein Interactions

**Figure 11:**
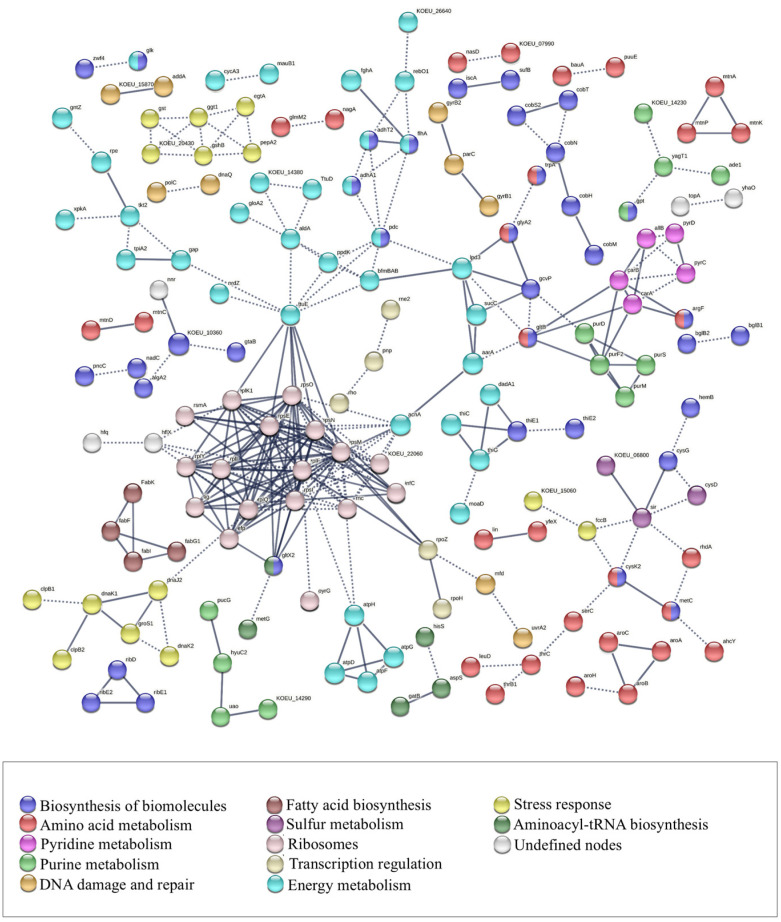
Protein–protein interaction network map (INM) for significant proteins of *K. europaeus*, those with a PPI *p*-value < 0.05, using the STRING v12.0 database (https://string-db.org, accessed on 2 March 2026). Proteins were represented as nodes in the networks, while interactions among them were shown by edges, the thickness of which indicates the strength of each interaction. Nodes with the same color were grouped according to the annotations of the proteins according to the UniProt and KEGG databases.

**Figure 12:**
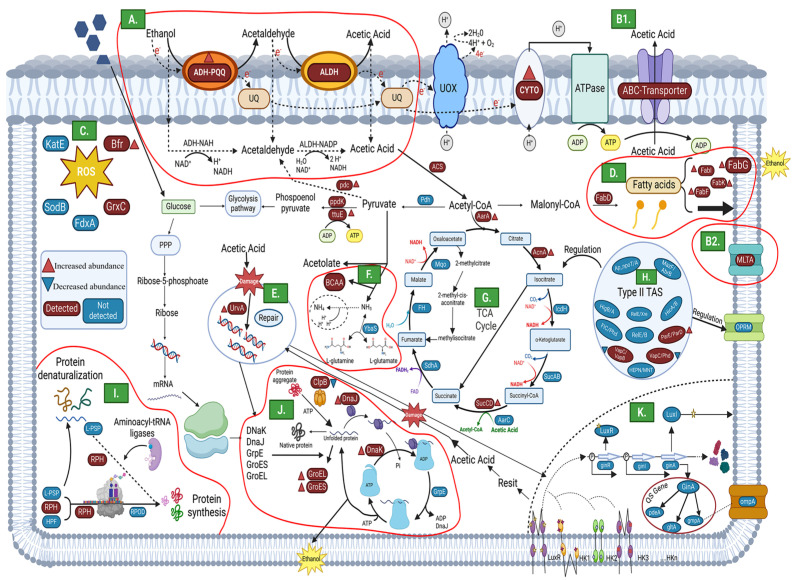
Metabolic pathways and adaptation mechanisms that may allow AAB to survive under the working conditions applied, characterized by a progressive increase in acidity. Red symbols indicate proteins experimentally detected in this study, whereas blue symbols indicate proteins included in the pathway scheme but not detected in the present dataset. Red triangles indicate proteins with increased abundance, and blue triangles indicate proteins with decreased abundance. (**A**) Respiratory chain in the oxidation of ethanol to acetic acid. (**B1**,**B2**) Membrane regulation mechanisms for acetic acid tolerance. (**C**) Enzymatic defense system against reactive oxygen species (ROS). (**D**) Fatty acid biosynthesis and changes in membrane composition. (**E**) DNA damage and repair. (**F**) Branched-chain amino acid metabolism. (**G**) Tricarboxylic acid (TCA) cycle. (**H**) Dependence on type II toxin–antitoxin systems. (**I**) Protein synthesis. (**J**) DnaK-DnaJ-GrpE chaperone system. (**K**) Quorum sensing (QS) system. Image created with BioRender. Campos-Vázquez, C. (2026) https://app.biorender.com/illustrations/6385eeab2f680565f511ba4a?slideId=db65160b-6510-4b72-a7f0-c926c7b98369 (accessed on 2 March 2026).

In order to determine the function of the main proteins identified in key metabolic pathways and processes, an analysis of protein–protein interactions was carried out. Given its predominance throughout the acetification process and the high number of proteins identified and assigned to this species, *K. europaeus* was selected as the model for this analysis. The focus on a single, highly represented AAB species allowed for the construction of a well-resolved functional protein network, facilitating the interpretation of the molecular mechanisms involved in acid stress adaptation in this species, which can serve as a reference framework for understanding the relevant functional processes in the microbial ecosystem of vinegar-making. Figure 11 presents the protein–protein interaction map generated from the proteome of *K. europaeus*, considering a total of 373 proteins that showed significant differences through the acetification process (see Table S5, Supplementary Materials). The analysis was carried out using the STRING v12.0 database (https://string-db.org, accessed on 2 March 2026), allowing the identification of functional networks and the prediction of associations among proteins. Based on the results obtained in the protein–protein interaction analysis, Figure 12 then summarizes the main routes and mechanisms of molecular adaptation that could allow AAB to survive under acidity conditions of submerged cultures.

Analysis of the protein–protein interaction for *K. europaeus* revealed a predominance of energy metabolism-related proteins (light blue, Figure 11), indicating that resistance to highly acidic environments requires considerable energy cost [35]. Key proteins associated with glycolysis and gluconeogenesis were identified, as well as components involved in ethanol oxidation metabolism, including two subunits of alcohol dehydrogenase (adhA1 and adhT2) and the protein flhA. ADH-PQQ enzymes are responsible for the oxidation of ethanol to acetaldehyde and, through acetification, it is usual to find them abundantly [37,40] (Figure 12A). During ethanol oxidation, electrons are generated and used in the respiratory chain. Ubiquinone oxidase (UOX) uses these electrons to reduce intracellular oxygen to water [3]. At the same time, the electrons lost in the process can be absorbed by coenzyme Q in the membrane, and the reduced coenzyme Q is subsequently oxidized by cytochrome oxidase (CYTO), contributing to the redox balance of the cell and the energy generation [3,46].

In addition, TCA cycle proteins (aarA, sucC, lpd3, gcvP, gltB, acnA) were detected (light pink, Figure 11), linking energy metabolism and protein synthesis. Three key genes (*aarA*, *aarB*, *aarC*) are central to acid resistance in AAB [47,48]. These genes support the metabolic adaptability of AAB to acid stress. AarA encodes citrate synthase, crucial for maintaining homeostasis under highly acidic conditions [35,37,40,49]. An increased abundance of proteins related to TCA enzymes has been reported, with aconitase (AcnA) showing the greatest increase [5]. The ribosomal protein cluster forms a complex structure located in the central left region of the network (light pink, Figure 11). This group constitutes one of the most prominent categories, indicating an intense modulation of protein synthesis in response to the cellular energy state. During acetification, the protein biosynthesis is one of the most important metabolic pathways in AAB to replenish cellular materials lost due to sudden changes that occur in the cycle [4,10,13,20].

On the other hand, the next most represented functional groups correspond to proteins related to the biosynthesis of biomolecules (dark blue, Figure 11) and the biosynthesis and metabolism of amino acids (red, Figure 11), reflecting the importance of these processes in the metabolic adaptation of *K. europaeus*. Amino acids constitute an important group of protective against oxidative damage caused by ethanol [40]. The oxidation of branched-chain amino acids (BCAA) (Figure 12F) generates metabolic energy and serves as a precursor in the synthesis of other amino acids, during catabolic states [5]. In this context, the deamination process, a well-documented strategy in several bacterial groups, involves the conversion of L-glutamine into L-glutamate, a reaction catalyzed by the enzyme glutaminase (such as YbaS) [37]. Furthermore, the breakdown of BCAAs releases ammonium ions (${NH}_{4}^{+}$), which contributes to the partial neutralization of the acids accumulated in the final stages of fermentation and helps to maintain the intracellular pH balance, favoring resistance to acetic acid [19,37,50,51].

Two distinct subgroups were identified within the stress response category (yellow, Figure 11):

1. One group, not interconnected with the rest of the network, located in the upper left corner, involved in glutathione metabolism and oxidative stress response. This group is made up of GTS, GGT1, EGTA, PEPA2, GSHB, and KOEU_20430, key proteins in cellular protection against reactive oxygen species (ROS) (Figure 12C) generated during ethanol oxidation. Under continuous aeration conditions, oxidative reactions can lead to the formation of toxic compounds, as well as the accumulation of ROS within the cytoplasm of AAB, which can affect their viability and cellular functioning. ROS can affect multiple cellular components, causing lipid peroxidation, protein oxidation, and alterations in DNA through genetic modifications, which can ultimately cause severe cellular damage or even cell death [37,40,52].
2. A group highly interconnected with the ribosomal protein cluster, located in the lower left corner, composed of proteins from the multichaperone system DnaK-DnaJ-GrpE and Clp (Figure 12J). This connection suggests a strong relationship between stress response and regulation of protein synthesis, which could indicate a strategy of *K. europaeus* to maintain protein functionality under conditions of high acidity. Heat shock proteins (HSP), known for their role in cell protection, play a key role in ensuring the correct folding of proteins synthesized under unfavorable conditions and preventing their intracellular denaturation [10]. Molecular chaperones have been widely identified within this protection system for their ability to prevent protein denaturation and facilitate refolding in stressful situations [37,53]. The quantification of these chaperones has been found to be significantly increased in different studies carried out under acid stress conditions [4,6,40,54]. The heat shock proteins GroEL and GroES also play an essential role in acetic acid fermentation [37]. It has been demonstrated that over-quantification of the *groEL* and *groES* genes in *A. pasteurianus* NBRC 3283 improves the resistance of the strains to adverse conditions, including high content of acetic acid, ethanol, and high temperatures [55]. This study found that the dnaK-dnaJ-grpE were found to be associated with proteins with increased abundance, which suggests an increase in the activity of these chaperones in response to acid stress. In contrast, it was observed that clpB has a decrease in abundance, which could indicate a lower need for processing protein aggregates under these specific conditions.

In addition, small groups of proteins involved in the biosynthesis of fatty acids (brown, Figure 11) were identified. Modifications in the composition of fatty acids in the cell membrane (Figure 12D) are an essential response of AAB to adapt to stressful environments. In our study, several proteins related to the biosynthesis of fatty acids were detected, such as FabK, FabF, FabI, and FabG1, as well as others associated with purine (light green, Figure 11) and pyrimidine metabolism (dark pink, Figure 11). The decreased abundance of FabD and FabG proteins, involved in the synthesis and elongation of fatty acids, has been observed under acetic acid stress conditions in *A. pasteurianus* [13,37,40,56]. In the group related to DNA damage and repair (Figure 11, orange) (Figure 12E), the presence of UvrA2, a key protein in the repair of DNA lesions, stands out. In AAB, UvrA is responsible for repairing the damage caused by exposure to acetic acid by nucleotide excision repair systems [57].

In the present study, it has been found that the protein TtuE, a pyruvate kinase, had an increase in abundance, being one of the central proteins in the protein–protein interaction network of *K. europaeus*, linked to ribosomal proteins, PPDK (pyruvate phosphate dikinase), and energy metabolism proteins, including those involved in the ethanol metabolism. It has been reported that Pyk, the classical pyruvate kinase in other Acetobacteraceae members, has a decreased abundance during semi-continuous fermentation, while PPDK quantification increases, indicating an inhibition of the glucose metabolism [3]. It has been demonstrated that the Embden-Meyerhof-Parnas (EMP) pathway is inoperative in certain *Acetobacter* spp., due to the absence of the phosphofructokinase enzyme. Without this essential component, the classic glycolysis pathway cannot operate, forcing the cell to use other pathways [58]. The increased abundance of TtuE in our study suggests that this enzyme could play a compensatory role against the low quantification of Pyk, allowing the conversion of phosphoenolpyruvate (PEP) to pyruvate and ensure the availability of ATP for cellular functions. Further studies are needed to elucidate the role of this protein in the metabolism of *K. europaeus*.

AAB have evolved type II toxin–antitoxin systems (TAS) (Figure 12H) to regulate growth and enhance adaptation to acid stress. These systems, composed of a stable toxin and an unstable antitoxin, modulate gene expression under adverse conditions. Toxins inhibit protein synthesis or degrade RNA, while antitoxins neutralize their activity [40,59,60,61]. In our study, VapC was identified, involved in the VapC/VapB and VapC/Phd pairs, whose quantity had a decrease in abundance. On the other hand, the ParE toxin, associated with the ParE/ParD pair, showed an increase in abundance. Quorum sensing (QS) signaling molecules and regulatory genes have been identified in several AABs, including *Gluconacetobacter diazotrophicus* and *K. xylinus* (Figure 12K) [40,62,63]. Although no QS proteins were detected in this study, this system is well-characterized in industrial microorganisms, such as *K. intermedius* [64]. In this species, genes such as *ginI* and *ginR* have been identified, which regulate the production of N-acyl-homoserine lactones (AHLs) and the activation of genes such as *gltA*, *pdeA*, and *gmpA*, which are involved in the metabolic regulation and production of acetic acid [40]. AAB have developed membrane transport mechanisms (Figure 12B1,B2) to regulate the intracellular acetic acid level and maintain homeostasis in high-acidity environments. The ABC transporter plays a fundamental role in the regulation of acetic acid production, whose concentration progressively increases during the fermentation phase, avoiding its inhibition by excessive [19,34,37]. On the other hand, by contributing to the maintenance of the peptidoglycan layer under these extreme conditions, MltA provides additional evidence of the importance of the cell membrane as a key mechanism for resistance to acid stress [5,10,19].

### 3.7. Limitations and Future Perspectives

Although this study provides valuable insights into protein abundance changes during the acetification process, some limitations inherent to the proteomic approach employed should be considered. The analysis was performed using a bottom-up LC-MS/MS strategy, in which proteins are identified and inferred from peptides generated after enzymatic digestion. While this approach enables broad proteome coverage, it does not allow a comprehensive characterization of the different proteoforms arising from a single gene, which may result from post-translational modifications, proteolytic processing, or sequence variants. Consequently, the identified proteins mainly represent inferred canonical forms, and the actual diversity of proteoforms present in the system may be underestimated.

Despite these limitations, the applied approach provides a robust overview of protein abundance dynamics during the acetification process. Future studies integrating complementary strategies, such as top-down proteomics or targeted analyses of specific post-translational modifications, could contribute to a more detailed characterization of proteome complexity in this system.

## 4. Conclusions

This study provides a comprehensive quantitative metaproteomic characterization of the microbial community involved in the acetification of Verdejo wine vinegar under submerged culture conditions. The results confirm the strong dominance of acetic acid bacteria from the family Acetobacteraceae, particularly *Komagataeibacter europaeus*, which was responsible for most of the detected proteins and concentrated on most of the metabolic activity throughout the process. The proteomic profiles revealed dynamic changes in protein abundance across the different stages of the acetification cycle, indicating a progressive metabolic reprogramming as ethanol decreases and acidity increases. Functional analyses showed that the main adaptive responses are associated with energy metabolism, amino acid biosynthesis, maintenance of cellular homeostasis, and mechanisms related to acid stress tolerance. The protein–protein interaction analysis further highlighted the importance of pathways related to ethanol oxidation, central metabolism, stress response systems, and membrane-associated processes that support cellular stability under highly acidic conditions. Overall, these findings improve the understanding of the molecular strategies that allow acetic acid bacteria to adapt and remain metabolically active during industrial vinegar production. This knowledge contributes to a deeper understanding of the functional ecology of acetification and may support future strategies aimed at optimizing fermentation performance and improving process stability in submerged vinegar production systems.

## Data Availability

The mass spectrometry proteomics data have been deposited in the ProteomeXchange Consortium via the PRIDE partner repository with the dataset identifier PXD071539.

## Supplementary Materials

The following supporting information can be downloaded at: https://www.mdpi.com/article/10.3390/proteomes14020027/s1. Table S1. Complete list of all valid proteins identified in the metaproteomic analysis. For each protein, the corresponding taxonomic assignment (organism), UniProt protein description, protein name, and gene name according to the UniProt database are provided. Table S2. List of protein compositions within the four clusters constituting the heatmap. Each cluster comprises all metaproteome common proteins grouped according to abundance patterns and includes detailed information. Table S3. List of Tukey’s post hoc test results for pairwise comparisons among fermentation times. Table S4. List of proteins identified from the entire metaproteome that showed significant differences in abundance during the acetylation process, classified according to their differential abundance (with increased/decreased abundance), the associated genes and the organism of origin. Table S5. List of differentially significant proteins of *Komagataeibacter europaeus* that form the protein–protein interaction network generated using the STRING v12.0 database, including associated genes and identification of the organism of origin.
